# Clinical Features and T‐Cell Repertoire of Chronic Myeloid Leukemia Patients Who Attempt Discontinuation of Tyrosine Kinase Inhibitors: The ISAC‐TFR Study

**DOI:** 10.1002/cam4.71142

**Published:** 2025-08-11

**Authors:** Hiroshi Ureshino, Yukio Nakamura, Yemima Budirahardja, Iwao Nozawa, Keisuke Kidoguchi, Kazuharu Kamachi, Shinya Kimura

**Affiliations:** ^1^ Division of Hematology, Respiratory Medicine and Oncology, Department of Internal Medicine Faculty of Medicine Saga University Saga Japan; ^2^ Department of Drug Discovery and Biomedical Sciences Faculty of Medicine Saga University Saga Japan; ^3^ Repertoire Genesis Inc. Osaka Japan

**Keywords:** T‐cell immunology, T‐cell receptor repertoire, treatment‐free remission, tyrosine kinase inhibitorchronic myeloid leukemia

## Abstract

**Background:**

While tyrosine kinase inhibitor (TKI) discontinuation is an established therapeutic goal, up to 60% of patients relapse after the first attempt. The feasibility of a second or third attempt at TKI discontinuation remains uncertain. Immune surveillance, particularly T‐cell and natural killer (NK) cell responses, may influence treatment‐free remission (TFR), although no definitive biomarkers for predicting sustained TFR have been identified.

**Methods:**

This retrospective study included 57 chronic myeloid leukemia (CML) patients who attempted TKI discontinuation. Clinical outcomes after the first, second, and third TFR attempts were analyzed, and T cell receptor (TCR) and B‐cell receptor (BCR) repertoire analyses were conducted on peripheral blood samples from 14 patients to investigate the immune landscape associated with TFR.

**Results:**

TFR1 at 1 year was 67.9% (95% confidence interval [CI], 53.9%–78.4%). Sixteen patients attempted a second discontinuation, achieving a 1‐year TFR2 rate of 31.2% (95% CI, 11.4%–53.6%). Patients maintaining *BCR::ABL1* mRNA levels below MR^4.5^ at 3 months post‐TKI discontinuation had a significantly lower risk of relapse (HR, 0.099; 95% CI, 0.012–0.829; *p* = 0.033). TCR repertoire analysis did not reveal distinct clonal expansions of T cells; however, a significant age‐related decline in T‐cell diversity was observed.

**Conclusion:**

T‐cell immunity in CML patients who have achieved a deep molecular response (DMR) may closely approximate that observed in healthy individuals.

## 1 Introduction

Over the past two decades, ABL1 tyrosine kinase inhibitors (TKIs) have led to marked improvements in the overall survival outcomes of patients with chronic myeloid leukemia in the chronic phase (CML‐CP) [1]. Several TKI discontinuation trials show that approximately 40%–80% of patients who achieve a durable deep molecular response (DMR) achieve sustained molecular remission even after TKI discontinuation [2, 3, 4, 5, 6, 7]; therefore, TKI discontinuation is considered a feasible therapeutic goal for patients with CML‐CP. However, inconsistent data generated from different study designs means that no definitive biomarkers of successful treatment‐free remission (TFR) have been identified. To establish reliable prognostic factors for TFR, it is essential to identify patients at high risk of molecular relapse. Several lines of evidence demonstrate that CML responds well to immunotherapy, including treatments such as interferon‐α, allogeneic hematopoietic stem cell transplantation, and donor lymphocyte infusion [8]; however, even if PCR no longer detects *BCR::ABL1* mRNA, a small number of CML cells are thought to remain. These findings underscore the requirement that the host immune system prevents CML relapse, emphasizing the pivotal role of cancer immunosurveillance in maintaining molecular remission following TKI discontinuation in patients with CML.

Natural killer (NK) cells are pivotal cells within the innate arm of the immune system, and T lymphocytes (T cells) play a critical role in adaptive immunity in mammals; both of these cell types make a significant contribution to immune defense against pathogens or cancers [9, 10]. Both NK and T‐cell‐mediated immunity play crucial roles in the maintenance of TFR in patients with CML [3, 11, 12, 13]. Previously, we reported that allelic polymorphisms of the killer immunoglobulin‐like receptor (KIR), a representative NK cell surface molecule regulating NK cell activation, are associated with treatment responses and the achievement of TFR in patients with CML [14, 15]. In addition, analysis of the T‐cell receptor (TCR) repertoire in patients with adult T‐cell leukemia demonstrated clonality of T cells at a single cell level [16]. A previous study identified T cells that suppress CML in patients who maintain TFR^11^, but overall, the data remain insufficient.

Approximately 20%–60% of patients who attempt a first TKI discontinuation experience molecular relapse. After resumption of TKI therapy, the majority regain durable molecular remission. Although these patients were thought to require lifelong TKI treatment, recent studies have examined the possibility of achieving TFR after a second attempt at TKI discontinuation. Indeed, studies confirmed that a second attempt at TKI discontinuation might be feasible, even after the first attempt failed [17, 18, 19, 20]; however, a second attempt at TKI discontinuation may not be the standard therapeutic strategy for CML.

Here, we retrospectively evaluated clinical outcomes after a first TFR failure in patients with CML‐CP to clarify the feasibility of a second or third attempt. Furthermore, we conducted TCR and B‐cell receptor (BCR) repertoire sequencing during the ISAC‐TFR study approved by the Institutional Review Board of Saga University (**protocol No. 2021‐05‐02**; T‐cell **I** mmunity and Tumor‐ **S** pecific **A** ntigens **C** ontribute to **T** reatment‐ **F** ree **R** emission following tyrosine kinase inhibitor discontinuation in chronic myeloid leukemia) to identify epitope motifs recognized by the TCR to achieve TFR.

## 2 Materials and Methods

### 2.1 Patients

Patients with Philadelphia chromosome‐positive CML‐CP (*n* = 75) who attempted discontinuation of TKIs following achievement of a durable DMR after TKI treatment at Saga University Hospital (Saga, Japan) between April 2002 and October 2023 were enrolled. CML was diagnosed according to the World Health Organization classification of myeloid neoplasms and acute leukemia. Baseline patient characteristics (age, sex, complete white cell counts, blast counts, percentage of eosinophils and basophils, hemoglobin level, platelet counts, molecular diagnosis (*BCR::ABL1* mRNA transcript level), and spleen size) were obtained from hospital records. The final follow‐up date was 30 September 2024. All clinical data were reviewed by two expert hematologists (HU and SK). The study protocol was approved by the institutional review board of Saga University (**protocol No. 2024‐10‐01**). All procedures involving human participants were conducted in accordance with the ethical standards of the institutional and/or national research committees, and in accordance with the Declaration of Helsinki. Informed consent was obtained using the opt‐out method; information related to the research study, including the aims, use of specimens, and the opportunity to opt out, was made public, and no patients objected to inclusion.

### 2.2 Definition of Molecular Responses and Molecular Relapse

Molecular responses were defined according to *BCR::ABL1* mRNA transcript levels, as measured by real‐time quantitative polymerase chain reaction (qPCR). The transcription‐mediated amplification (TMA) method and/or the international scale (IS) were used. The TMA method was approved in Japan prior to the introduction of the IS, and demonstrates a high correlation with European standard methods based on qPCR [21]. A major molecular response (MMR) was defined as a *BCR::ABL1* mRNA transcript level of < 50 copies/0.5 mg RNA (TMA) or ≤ 0.1% (IS). MR^4.0^ was defined as undetectable *BCR::ABL1* mRNA (TMA) or ≤ 0.01% (IS). MR^4.5^ was defined as ≤ 0.0032% (IS). DMR was defined as MR^4.0^ or deeper, and molecular relapse was defined as loss of the MMR [22].

### 2.3 Analysis of the TCR and BCR Repertoire

This study describes a novel high‐throughput sequencing method developed to analyze the TCR α/β and BCR repertoires. The approach combines adaptor‐ligation‐mediated PCR (AL‐PCR) with 454 DNA sequencing technology to minimize amplification bias typically observed in conventional multiplex PCR methods. Peripheral blood mononuclear cells (PBMCs) were obtained from patients with written informed consent, and total RNA was extracted using the RNeasy Lipid Tissue Mini Kit (Qiagen, Hilden, Germany). One microgram of total RNA was converted to complementary DNA (cDNA) with Superscript III reverse transcriptase (Invitrogen, Carlsbad, CA) and a poly (T) primer containing a NotI site. Double‐stranded cDNA was generated with *E. coli* DNA polymerase I, ligase, and RNase H, blunt‐ended using T4 DNA polymerase, and ligated to a double‐stranded adaptor (P10EA/P20EA). After adaptor ligation, a two‐step PCR was performed using adaptor and TCR/BCR constant region‐specific primers to achieve unbiased amplification of all receptor transcripts. Amplicons were purified and subjected to emulsion PCR using the amplicon mixtures with GS Junior Titanium emPCR Lib‐L kit (Roche 454 Life Sciences, Branford, CT) and sequencing using the Roche 454 sequencing system. A barcoding system with fusion tag primers enabled sample multiplexing. Sequence reads were demultiplexed, quality‐filtered, and analyzed using a custom repertoire analysis platform developed by Repertoire Genesis Inc. (Osaka, Japan) [23, 24], which aligns reads to the IMGT database (http://www.imgt.org) and identifies gene usage (TRAV/TRAJ or TRBV/TRBJ), CDR3 regions, and clonal expansions. Diversity indices such as Simpson and Shannon–Weaver were also calculated. All TCR and BCR repertoire analyses were performed by Repertoire Genesis Incorporation, as previously described [23, 24]. All procedures were conducted in accordance with the Declaration of Helsinki and were approved by the Saga University Institutional Review Board (**Protocol No. 2021‐05‐02**).

### 2.4 Statistical Analysis

The cumulative incidence of TFR was calculated using the Kaplan–Meier method, and differences were analyzed using the log‐rank test. A Cox proportional hazard model was used to evaluate TFR, and two‐sided *p* values < 0.05 were considered statistically significant. The Mann–Whitney *U* test was used to determine statistically significant differences between two groups or variables. All statistical analyses were performed using EZR (Saitama Medical Center, Jichi Medical University), a graphical user interface for R.

## 3 Results

### 3.1 Patient Characteristics in First TKI Discontinuation

**FIGURE 1:**
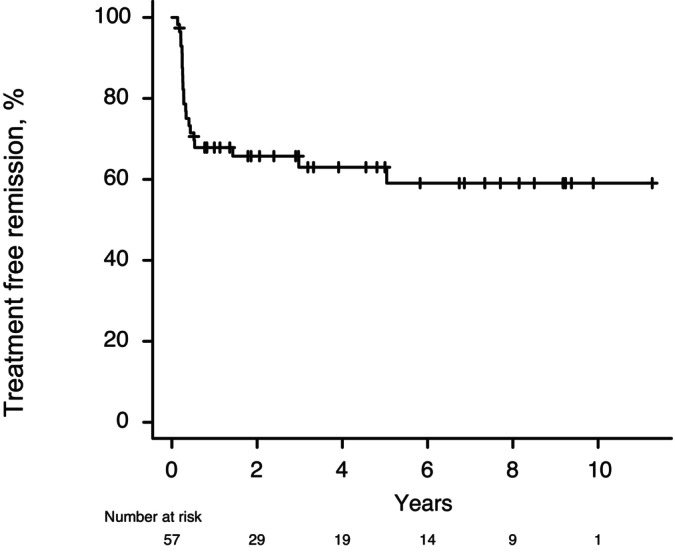
Treatment free remission (TFR) in patients with chronic myeloid leukemia after a first attempt at tyrosine kinase inhibitor discontinuation.

A total of 75 CML‐CP patients (45 male and 30 female) discontinued TKIs for the achievement of TFR. Out of 75 patients, 18 resumed TKI after experiencing DMR loss and were therefore excluded from the analysis. A total of 57 patients (37 male and 20 female) discontinued TKIs. The median age was 58 years (interquartile range (IQR), 50–68 years). Of these, 6, 13, and 34 patients had a high, intermediate, or low Sokal risk score, respectively (four patients had missing data). The front‐line TKI was imatinib in 14 cases, dasatinib in 33, nilotinib in nine, and bosutinib in one. The TKI used before the first discontinuation was imatinib in seven patients, dasatinib in 36, nilotinib in eight, bosutinib in five, and ponatinib in one. Molecular remission status at the first attempt at attaining TFR (i.e., TFR1) was MR^4.0^ for 13 patients, MR^4.5^ for 28, and undetectable residual disease (UMRD) for 16. The median duration of TKI treatment and the DMR were 59.0 months (IQR, 42.8–94.4 months) and 39.3 months (IQR, 32.0–57.6 months), respectively. The median follow‐up time for those in TFR1 was 51.5 months (IQR, 24.1–95.2 months). Of the 57 patients analyzed, TFR1 at 1 year was 67.9% (95% confidence interval [CI], 53.9%–78.4%), 63.0% (95% CI, 48.4%–74.5%) at 3 years, and 63.0% (95% CI, 48.4%–74.5%) at 5 years, respectively (Figure 1). The median time to molecular relapse was 3.2 months (IQR, 2.9–5.1 months). Three patients experienced late relapse (≥ 1 year) at a median of 1087 days; these patients tended to be younger than patients with early molecular relapse (46 vs. 58 years, *p* = 0.207). The detailed clinical characteristics are summarized in Table 1. Univariate analysis of the clinical characteristics of patients in TFR1 (i.e., sex, age, Sokal risk score, front‐line TKI, TKI at discontinuation, IS at TKI discontinuation, DMR time, and TKI treatment duration) did not identify any factor as a significant prognostic variable for a lower likelihood of MR (Table 2).

**TABLE 1:** Patient characteristics (*n* = 57).

	Median, *n*	IQR, %
Age at stop	58	50–68
Gender		
Male	37	64.9%
Female	20	35.1%
Sokal risk score		
High	6	10.5%
Intermediate	13	22.8%
Low	34	59.6%
Missing	4	7.0%
Front‐line TKI		
Imatinib	14	24.6%
Dasatinib	33	57.9%
Nilotinib	9	15.8%
Bosutinib	1	1.8%
TKI at stop		
Imatinib	7	12.3%
Dasatinib	36	63.2%
Nilotinib	8	14.0%
Bosutinib	5	8.8%
Ponatinib	1	1.8%
IS at stop		
MR^4.0^	13	22.8%
MR^4.5^	28	49.1%
UMRD	16	28.1%
DMR duration (months)	39.3	32.0–57.6
TKI treatment duration (months)	59.0	42.8–94.4
Re‐DMR time from relapse (days) (*n* = 21)	122	97–141
Postrelapse selected TKI (*n* = 21)		
Same TKI	11	53.8%
Changed TKI	10	46.2%

*Note:* 59.0 months (IQR, 42.8–94.4 months).

Abbreviations: DMR, deep molecular response; IS, international scale; TKI, tyrosine kinase inhibitor; UMRD, undetectable minimal residual disease.

**TABLE 2:** Univariate analysis for the achievement of treatment‐free remission.

Variable	HR	95% CI	*p*
Gender			
Female	Ref		
Male	0.727	0.306–1.727	0.471
Age ≥ 58‐year‐old	0.810	0.344–1.908	0.630
Sokal risk			
High	Ref		
Int	2.879	0.336–24.66	0.335
Low	2.495	0.324–19.23	0.380
Missing	5.435	0.564–52.35	0.143
Front‐line TKI			
Imatinib	Ref		
Bosutinib	11.80	1.150–121.0	0.038
Dasatinib	2.949	0.849–10.250	0.089
Nilotinib	1.162	0.194–6.963	0.870
TKI at stop			
Imatinib	Ref		
Bosutinib	3.232	0.706–14.81	0.131
Dasatinib	0.835	0.236–2.960	0.780
Nilotinib	0.613	0.102–3.676	0.592
Ponatinib	< 0.001	0.000–Inf	0.998
IS at TKI stop			
MR^4.0^, MR^4.5^	Ref		
UMRD	0.809	0.296–2.209	0.679
DMR time ≥ 39.3 months	1.101	0.467–2.594	0.827
Treatment duration ≥ 59.0 months	0.852	0.362–2.006	0.714

Abbreviations: DMR, deep molecular response; IS, international scale; TKI, tyrosine kinase inhibitor; UMRD, undetectable minimal residual disease.

### 3.2 Characteristics of Patients Who Attempted a Second TKI Discontinuation

**FIGURE 2:**
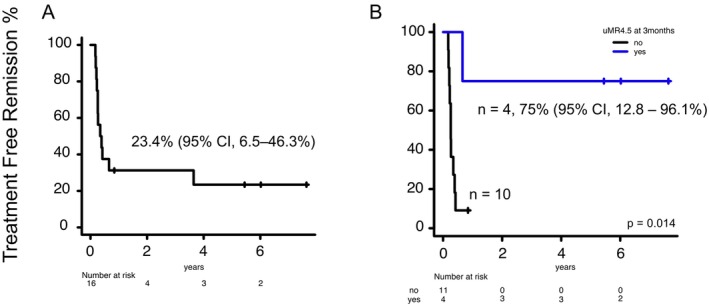
Treatment free remission (TFR) in patients with chronic myeloid leukemia after a second attempt at tyrosine kinase inhibitor (TKI) discontinuation (i.e., TFR2). TFR2, according to *BCR::ABL1* mRNA international scale status, at 3 months after TKI discontinuation.

Including patients who resumed TKI due to DMR loss, 39 of 75 patients were defined as molecular relapse. Sixteen of the 39 patients (41.0%) who failed TFR1 attempted a second TKI discontinuation (TFR2). The median age was 63 years (IQR, 60–67 years); nine patients were male, and one, four, and eight had a high, intermediate, or low Sokal risk score, respectively (three patients had missing data). The TKI at the TFR1 attempt was imatinib in five patients and dasatinib in 11, and the TKI at the TFR2 attempt was dasatinib in 12 patients, nilotinib in two, and bosutinib in two. Eight of the 16 patients changed TKI between the TFR1 attempt and the TFR2 attempt at the discretion of their primary physician due to failure of the TFR1 attempt. The median TKI treatment duration from TFR1 failure to the TFR2 attempt was 38.3 months (IQR, 35.5–56.0 months), the median time to a DMR from TFR1 failure was 113 days (83–173 days), and the median duration of a DMR was 35.5 months (IQR, 32.3–47.1 months). Molecular remission status at the TFR2 attempt was MR^4.0^ for two patients, MR^4.5^ for three, and UMRD for 11. The percentage of patients in TFR2 at 1 year was 31.2% (95% CI, 11.4%–53.6%), that at 3 years was 31.2% (95% CI, 11.4%–53.6%), and that at 5 years was 23.4% (95% CI, 6.5%–46.3%; Figure 2A). The median time to relapse at TFR2 was 3.2 months (IQR, 2.7–4.9 months). The detailed clinical characteristics of patients attempting TFR2 are summarized in Table 3.

**TABLE 3:** Characteristics of patients at the second attempt at treatment‐free remission.

	Median, n	IQR, %
Age at discontinuation	63	60–67
Sex		
Male	9	56.3%
Female	7	43.8%
Sokal risk score		
Low	8	50.0%
Intermediate	4	25.0%
High	1	6.3%
Missing	3	18.8%
Prior failed TKI		
Imatinib	5	31.3%
Dasatinib	11	68.8%
Same TKI at stop as prior failed TKI		
Same TKI	8	50.0%
Changed TKI	8	50.0%
TKI at discontinuation		
Dasatinib	12	75.0%
Nilotinib	2	12.5%
Bosutinib	2	12.5%
IS at discontinuation		
MR^4.0^	2	12.5%
MR^4.5^	3	18.8%
UMRD	11	68.8%
DMR duration (months)	35.5	32.3–47.1
TKI treatment duration from relapse (months)	38.3	35.5–56.0
Re‐DMR time from relapse (days)	113	83–173

Abbreviations: DMR, deep molecular response; IQR, interquartile range; IS, international scale; TKI, tyrosine kinase inhibitor; UMRD, undetectable minimal residual disease.

Although 23 of 39 patients (59.0%) did not attempt TFR2, almost all patients achieved a DMR or deeper (MMR, one; MR^4.0^, two; MR^4.5^, six; UMRD, 14) after receiving TKI treatment. One patient only achieved MMR (IS, 0.0132%) and is at the 1‐month mark after molecular relapse; further improvement is expected with continued TKI treatment. Notably, the median time to re‐DMR for patients who attempted TFR2 was significantly shorter than that for those who did not attempt TFR2 (80 days vs. 130 days, respectively; *p* = 0.001). Fourteen of the 16 patients were deeper than MR^4.5^ at the time of second TKI discontinuation. Univariate analysis of 14 patients was conducted to identify prognostic factors related to the second attempt TFR. The results showed that patients maintaining under MR^4.5^ at 3 months post‐discontinuation of TKI had a lower risk of relapse than those with levels above MR^4.5^ (HR, 0.103; 95% CI, 0.012–0.874; *p* = 0.037; Figure 2B). Three of 16 patients (18.8%) with TFR2 failure attempted a third TKI discontinuation (i.e., TFR3). Only one patient achieved TFR3 [25]. The detailed clinical characteristics of those who attempted a third discontinuation are summarized in Table S1.

### 3.3 Loss of a DMR After Long‐Term Maintenance of a DMR May Be a Risk Factor for MMR Loss

Patients who maintained TFR included those who lost a DMR temporarily. Five patients were identified, and all lost a DMR at a median of 105 days (IQR, 83–120), but either regained a DMR or maintained MMR without resuming TKIs (Figure S1), referred to as fluctuated patients [22]. By contrast, three patients who maintained a DMR long‐term (> 1 year) and then lost a DMR at a median of 861 days (IQR, 597–981 days) subsequently experienced loss of MMR (Figure S2). There was a significant difference between the groups with respect to the time until loss of a DMR (*p* = 0.036); thus, patients who lose a DMR after long‐term maintenance of a DMR may be at risk of late relapse.

### 3.4 Recent Treatment Choices for Patients Who Failed a TFR Attempt

Thirty‐four of the 75 patients experienced TFR failure, including TFR1, TFR2, and TFR3, and so continued to receive TKI treatment. Almost all achieved re‐DMR (32 of 34 patients, 94.1%) after molecular relapse, indicating that re‐attempting TFR is a feasible treatment strategy. Recently, a first‐in‐class STAMP (specifically targeting the ABL myristoyl pocket) inhibitor, asciminib, was approved for treatment of patients with CML‐CP who exhibited resistance or intolerance to two prior TKI therapies [26]. Most patients received asciminib (14 of 34 patients), and more than half (8 of 14 patients) achieved UMRD (Table S2). A male patient who failed a TFR2 attempt at 240 days after dasatinib discontinuation (**CML‐73**) regained a DMR approximately 1 month after resuming dasatinib. The patient experienced loss of a DMR after 20 months, but subsequently maintained a MMR for 18 months after dasatinib treatment. Notably, there was a rapid increase in the IS (84.1915%) during dasatinib treatment, and the T315I mutation was detected. Treatment was switched to ponatinib, resulting in a rapid reduction in the IS to 0.4707% within 3 months, indicating that ponatinib is effective for patients who harbor the T315I mutation.

### 3.5 T‐Cell Diversity Is Independent of TFR Status, but Decreases With Age in Patients With CML

**FIGURE 3:**
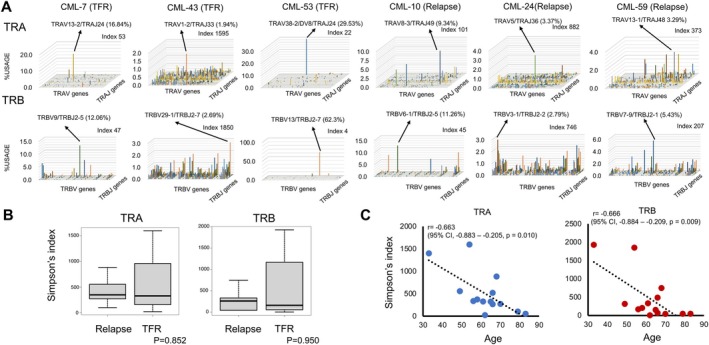
Clonal diversity and patterns of gene usage by T‐cell receptor (TCR) alpha (TRA) and beta (TRB) chains in patients with chronic myeloid leukemia (CML). Three‐dimensional analysis of TRA/TRB repertoires along x‐, y‐, and z‐axes show the TRAV/TRAJ, TRBV/TRBJ, and frequency percentage, respectively. Use of TRAV/TRAJ region genes and TRBV/TRBJ region genes in patients with CML (three treatment free remission (TFR) cases and three relapsed cases) (A). Simpson's index for TRA and TRB according to TFR status (B). Reductions in the TRA/TRB ratio with age (C).

We hypothesized that TFR patients have a higher number of CML‐specific T‐cell clones in order to eradicate leukemic cells; therefore, we conducted TCR repertoire analysis of patients who attempted TKI discontinuation. Fourteen patients were enrolled in the ISAC‐TFR study. Of these, five maintained TFR1 and nine experienced molecular relapse at the first attempt at TKI discontinuation. Two of nine relapsed patients achieved TFR2 at the time the blood samples were collected. Therefore, we categorized seven of the patients as TFR and eight as relapsed. Seven patients were male, and seven were female, and the median age at enrollment was 64 (IQR, 57–68) years. The median interval between TKI discontinuation and sample collection was 52.3 (IQR, 44.8–61.9) months. Analysis of the TCR‐α and TCR‐β chain (TRA/TRB) repertoires, focusing on expression levels of TRAV/TRAJ and TRBV/TRBJ region genes, identified skewed T‐cell clones (i.e., T cells with the same antigen specificity become abnormally dominant) in two TFR cases (i.e., CML‐7 and CML‐53); however, case CML‐43 did not exhibit such skewing. A similar pattern was observed in relapsed cases (CML‐10, CML‐24, and CML‐59; Figure 3A). To assess the diversity of the TCR repertoire, we calculated the mean copy number of unique sequence reads and calculated diversity indices, including Simpson's index. A decrease in Simpson's index would reflect the expansion of CML‐specific T‐cell clones. There was no significant difference in Simpson's index for the TRA and TRB repertoires between the TFR and relapsed cases (Figure 3B), suggesting comparable T‐cell diversity in these groups. We also analyzed the BCR to identify CML‐specific epitopes, and again found no differences in the diversity of IgM and IgG BCRs between TFR cases and relapsed cases (Figure S3A,B). Next, we investigated whether shared TCR clones were present among the five TFR1 cases, and found no shared TCR clones with 10 or more reads across all five TFR1 cases; however, TCR clones shared between two or more cases included 10 TRA clones and one TRB clone (Table S3). Of these, none were significant (i.e., exceeding 1%) in any of the shared pairs of cases. HLA class I TREM epitopes consist of 9‐mer peptides, with a central 5‐mer segment recognized by the TCR. Although we analyzed HLA class I‐binding TREMs, no common motifs specific to TFR cases were identified. Although one common TREM motif (___GILVT_) associated with *SLC34A1*, *MBOAT1*, and *ADM12* was detected in a TFR case (CML‐53)and a relapsed case (CML‐59), its biological significance warrants further investigation. Simpson's index for TRA and TRB was 330.0 (IQR, 267.5–546.0) and TRB 187.5 (IQR, 52.5–447.0), respectively. Notably, Simpson's index for TRA and TRB showed a negative correlation with patient age, indicating an age‐related reduction in T‐cell diversity (Figure 3C). The diversity of BCRs also tended to decrease with increasing age (Figure S3C).

## 4 Discussion

Here, we conducted a retrospective analysis of patients with CML‐CP who failed a first attempt at TKI discontinuation, and examined their TCR/BCR repertoires. The findings of a TFR1 rate of 67.9% (95% CI, 53.9%–78.4%) [2, 3, 4, 5, 6], and a TFR2 rate at 1 year of 31.2% (95% CI, 11.4%–53.6%) [17, 18, 19, 20], were similar to the previous TKI discontinuation studies, indicating that our patients were a representative cohort.

Male sex [2], long‐term TKI treatment, and DMR duration [27], deeper molecular remission status [28], immune cell activation status [11, 15] and a lower Sokal risk score [2] are favorable prognostic factors for TFR1 in patients with CML‐CP; however, we did not identify any of these as favorable prognostic factors for sustained TFR1. In our cohort, the majority of patients attempted treatment discontinuation with dasatinib. In TKI discontinuation studies utilizing dasatinib, such as the DADI and D‐STOP trials [3, 4, 29], no clinical parameters have been identified as prognostic factors for TFR. Therefore, the present study is likely to yield similar findings. Later molecular relapse at TFR1 (> 3 months) [17, 19] and sustained below MR^4.5^ within 3 months after the TFR2 attempt are associated with successful achievement of TFR2 [17, 20]. Our results also showed that loss of under MR^4.5^ within 3 months might predict molecular relapse, suggesting that the parameter may be a predictive marker for achievement of TFR2.

Despite more than half of the patients achieving a DMR after the first molecular relapse, a second attempt at TFR was not attempted. Several patients opted to continue TKI treatment due to the perceived risk of relapse and continued treatment without difficulty due to minimal experience of adverse events, as previously reported [30]; however, for the remaining cases, their medical records provided no clear reasons to deny the second TFR attempt. Notably, the decision to attempt a second TFR in cases that achieved early remission may reflect the confidence of the attending physicianabout the chances of success.

Almost all patients (32/34, 94.1%) re‐achieved DMR after molecular relapse, primarily after treatment with a second‐ or later‐generation TKI. Second‐generation TKIs are more potent than imatinib [31, 32, 33], and asciminib is even more potent than second‐generation TKIs in patients with newly diagnosed or resistant/intolerant CML [34, 35]. Ponatinib is particularly effective for patients carrying the T315I mutation [36]; indeed, our patient with the T315I mutation demonstrated a rapid response to ponatinib following relapse. These results suggest that improvements in TKIs have enabled more patients to achieve a DMR, making multiple TFR attempts a feasible treatment for patients with CML.

Fluctuations in *BCR::ABL* mRNA levels following TKI discontinuation have been reported previously [22], with some cases maintaining MMR or re‐entering a DMR. The present study shows that in patients who maintained a long‐term DMR, loss of a DMR warrants careful monitoring to detect potential loss of MMR. This underscores the importance of continued follow‐up, even in patients who sustain a DMR.

Immune surveillance may play a protective role in preventing relapse in CML patients after TKI discontinuation; indeed, we reported previously that NK cell immune responses are crucial for the maintenance of TFR [14, 15]. By contrast, a long‐lasting T‐cell immune response may play a critical role in maintaining TFR [12]. TCR and BCR repertoire analysis can identify T‐cell or B‐cell clones specific for pathogens or cancer cells [24]. TCR repertoire analysis of TRB genes showed that patients with CML have restricted CD4^+^ or CD8^+^ T cells [37], and one TFR case had long‐lasting memory T‐cell clones [12]. Unfortunately, common T‐cell clones could not be identified in TFR cases; however, T‐cell clones with strong clonality were observed in some cases, suggesting that some of these may represent CML‐specific T‐cell clones. Although the present study did not clarify the impact of T‐cell immunity on TFR in patients with CML, future research will be needed to clarify the relationship between T‐cell immunity and TFR. Nevertheless, the present study is considered a valuable contribution as it analyzes the TCR/BCR repertoire in TFR attempt CML cases.

Patients with CML have restricted CD4^+^ or CD8^+^ T‐cell clones, and reductions in TCR repertoire diversity with age may be associated with increased susceptibility to infectious disease [38]. In this study, we found that the diversity of TRA and TRB in CML patients was lower than that reported in healthy individuals, suggesting a possible increase in T‐cell clonality in CML patients; however, the median age of healthy individuals in previous studies was 31.5 years [24], whereas the median age in this study was 64 years. Given that a decline in diversity is associated with aging, this difference is most likely attributable to age‐related factors. T‐cell immunity in CML patients who have achieved a DMR may closely approximate that observed in healthy individuals.

A recent analysis of TCR repertoires has demonstrated that cytomegalovirus (CMV)‐specific T‐cell clonotypes expand uniquely during dasatinib treatment [39], suggesting the possibility of subclinical CMV reactivation. Another study showed that PR1‐specific TCRs were significantly expanded in CML patients compared to healthy controls [40]. Among various antigen‐specific TCRs, including viral and melanoma‐associated epitopes, the anti‐PR1 response was the most prominent in CML, particularly during dasatinib treatment. During TKI treatment, anti‐PR1 TCRs comprised a similar proportion of the repertoire in both the TFR and relapse patients. After TKI cessation, most large clones contracted significantly in the relapse patient, whereas this was less frequent in the TFR patient. These findings highlight distinctive immune dynamics and suggest a potential role for PR1‐specific T cells in influencing treatment outcomes in CML.

To summarize, a TFR2 attempt is a feasible strategy in selected patients with CML, with favorable outcomes associated with maintenance of MR^4.5^ within 3 months. However, we did not identify specific TCR clones that predict TFR.

## Author Contributions

**Hiroshi Ureshino:** conceptualization, methodology, investigation, software, data curation, formal analysis, validation, visualization, funding acquisition, writing – original draft, writing – review and editing, project administration. **Yukio Nakamura:** methodology, software, data curation, validation, investigation, formal analysis, project administration. **Yemima Budirahardja:** data curation, software, formal analysis, validation, methodology, investigation, project administration. **Iwao Nozawa:** software, methodology, validation, investigation, project administration, formal analysis, data curation. **Keisuke Kidoguchi:** data curation, supervision, writing – original draft, writing – review and editing. **Kazuharu Kamachi:** data curation, supervision, writing – original draft, writing – review and editing. **Shinya Kimura:** funding acquisition, writing – original draft, conceptualization, methodology, data curation, supervision, writing – review and editing.

## Conflicts of Interest

H.U. has received honoraria from Novartis. N.Y., B.Y., and I.N. are full‐time employees of Repertoire Genesis Inc. S.K. received honoraria from Pfizer, Otsuka Pharmaceuticals, Novartis, and Bristol‐Myers‐Squibb, as well as research funding from Pfizer, Bristol‐Myers‐Squibb, and Ohara Pharmaceuticals. The remaining authors declare no competing financial interests.

## Supporting information


**Data S1:** Supporting Information.

**Data S2:** Supporting Information.

**Data S3:** Supporting Information.

**Data S4:** Supporting Information.

**Data S5:** Supporting Information.

**Data S6:** Supporting Information.

## Data Availability

The data that support the findings of this study are available from the corresponding author upon reasonable request.
