# Supplementary material for: Clinical Features and T‐Cell Repertoire of Chronic Myeloid Leukemia Patients Who Attempt Discontinuation of Tyrosine Kinase Inhibitors: The ISAC‐TFR Study
Source: Cancer Med. 2025 Aug 11;14(15):e71142. doi: 10.1002/cam4.71142 (PMC12336671; doi:10.1002/cam4.71142)
Supplement: Supplementary file 2 — Data S2: Supporting Information. [file CAM4-14-e71142-s004.pdf]

Supplementary Figure 2

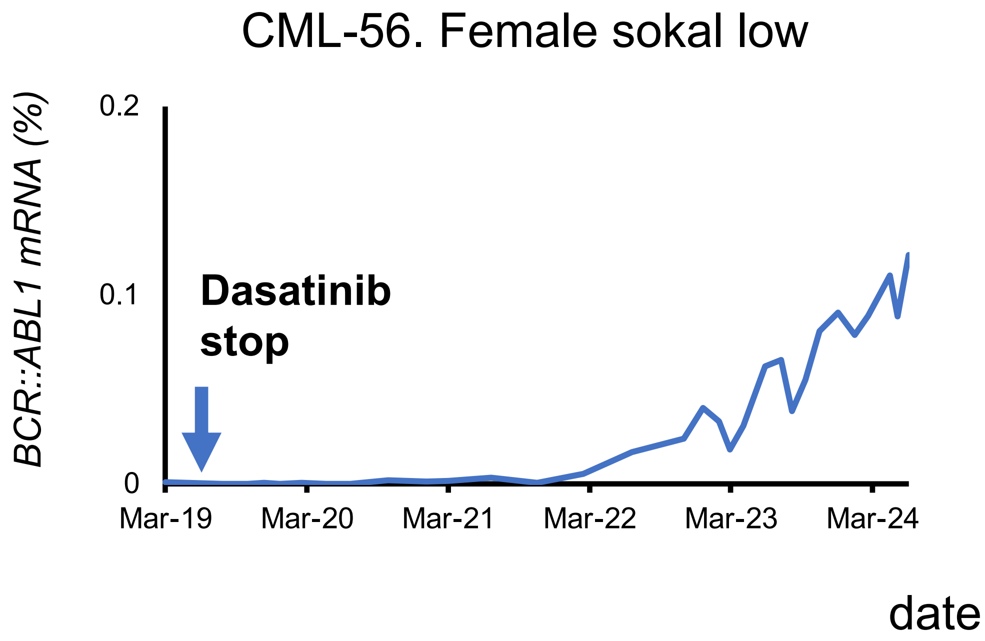

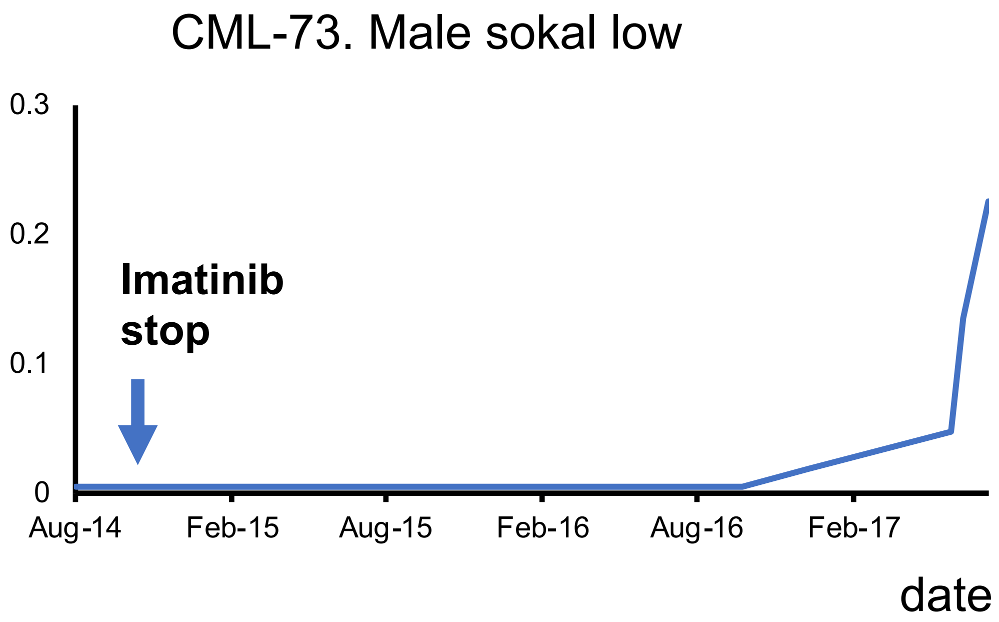

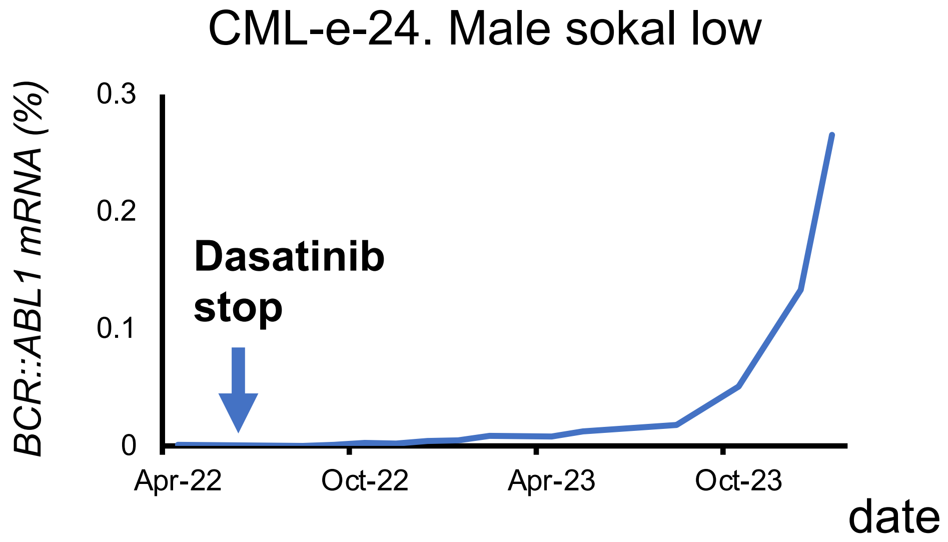

Figure S2. Longitudinal monitoring of *BCR::ABL1* transcripts in patients with chronic myeloid leukemia patients who discontinued tyrosine kinase inhibitors.
