# Supplementary material for: Clinical Features and T‐Cell Repertoire of Chronic Myeloid Leukemia Patients Who Attempt Discontinuation of Tyrosine Kinase Inhibitors: The ISAC‐TFR Study
Source: Cancer Med. 2025 Aug 11;14(15):e71142. doi: 10.1002/cam4.71142 (PMC12336671; doi:10.1002/cam4.71142)
Supplement: Supplementary file 3 — Data S3: Supporting Information. [file CAM4-14-e71142-s002.pdf]

Supplementary Figure 3

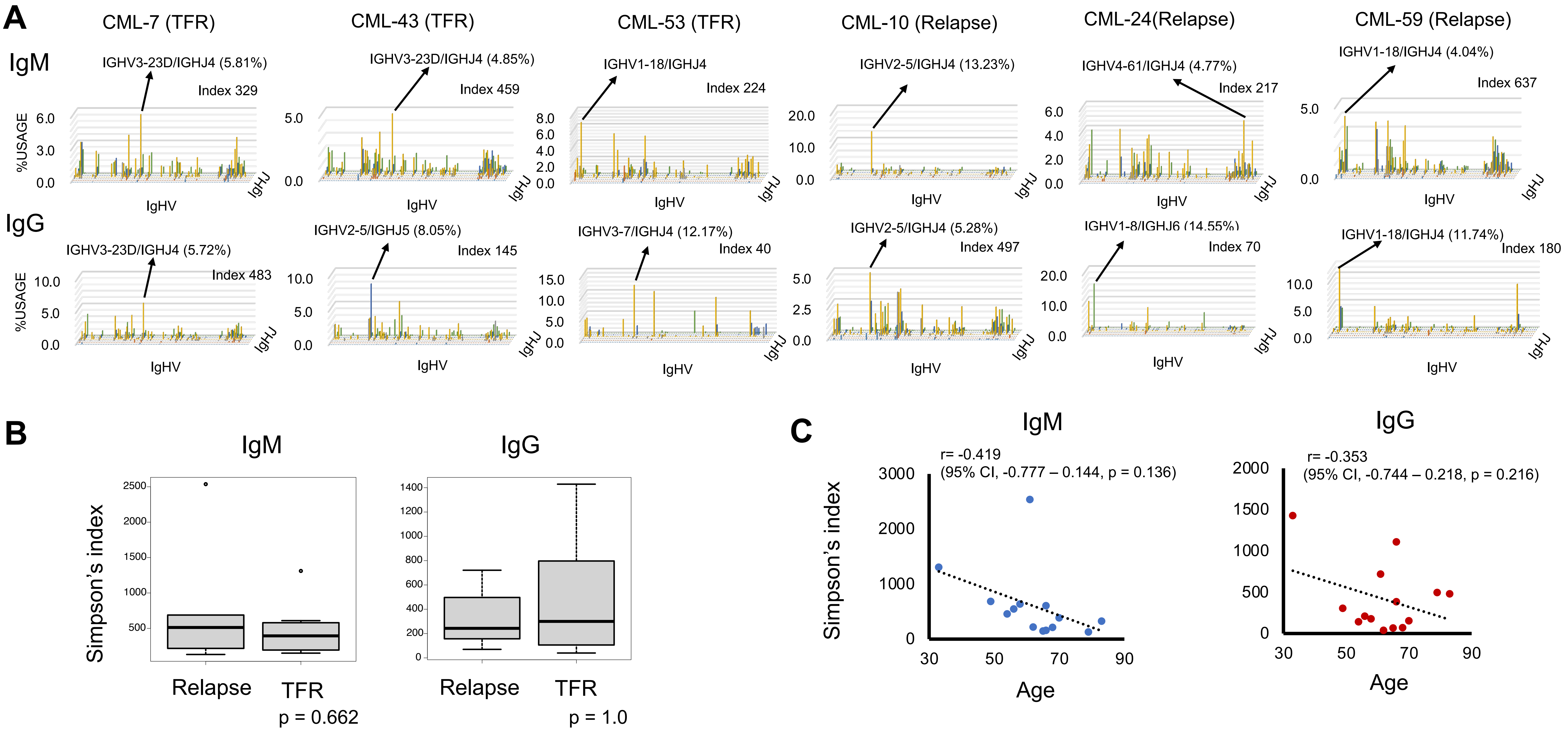

Figure S3. Clonal diversity and patterns of gene usage by B cell receptor (BCR) (IgM and IgG) in patients with chronic myeloid leukemia (CML). Three-dimensional analysis of TRA/TRB repertoires along x-, y-, and z-axes show the IgHV/IgHJ, and frequency percentage, respectively. Use of IgHV/IgHJ region genes in patients with CML (three treatment free remission (TFR) cases and three relapsed cases) (A). Simpson's index for BCR (IgM and IgG) according to TFR status (B). Reductions in the BCR ratio with age (C).
