# Supplementary material for: Clinical Features and T‐Cell Repertoire of Chronic Myeloid Leukemia Patients Who Attempt Discontinuation of Tyrosine Kinase Inhibitors: The ISAC‐TFR Study
Source: Cancer Med. 2025 Aug 11;14(15):e71142. doi: 10.1002/cam4.71142 (PMC12336671; doi:10.1002/cam4.71142)
Supplement: Supplementary file 4 — Data S4: Supporting Information. [file CAM4-14-e71142-s001.docx]

**Table S1. Characteristics of patients at the third attempt at treatment-free remission (TFR3)**

|  | 1 | 2 | 3 |
| --- | --- | --- | --- |
| Front-line TKI | Imatinib | Imatinib | Imatinib |
| First stop TKI (TFR1) | Imatinib | Imatinib | Imatinib |
| Sokal risk score | Int | Low | Unknown |
| Age at discontinuation (TFR1) | 60 | 64 | 58 |
| IS at discontinuation (TFR1) | MR^4.0^ | MR^4.0^ | MR^4.5^ |
| Duration of DMR (months, TFR1) | 79.3 | 45 | 38.9 |
| Duration of molecular relapse (days) | 123  (DMR loss) | 46  (DMR loss) | 92  (MMR loss) |
| Duration from relapse to achievement of DMR (days) | 59 | 54 | 92 |
| Second discontinuation TKI | Dasatinib | Nilotinib | Dasatinib |
| Age at discontinuation (TFR2) | 64 | 65 | 62 |
| IS at discontinuation (TFR2) | MR^4.0^ | MR^4.0^ | UMRD |
| Duration of DMR (months, TFR2) | 24.1 | 24.2 | 35.7 |
| Duration of molecular relapse (days) | 1331  (MMR loss) | 64  (MMR loss) | 95  (MMR loss) |
| Duration from relapse to achievement of DMR (days) | 35 | 182 | 120 |
| Third discontinuation TKI | Dasatinib | Nilotinib | Bosutinib |
| Age at discontinuation (TFR3) | 69 | 74 | 65 |
| IS at discontinuation (TFR3) | MR^4.5^ | UMRD | MR^4.5^ |
| Duration of DMR (months, TFR3) | 34.2 | 37.8 | 32.7 |
| Duration of molecular relapse (days) | 56  (MMR loss) | TFR | 133  (MMR loss) |
| Ongoing treatment | Dasatinib | no | Asciminib |
| Recent IS | MR^4.5^ | UMRD | MR^4.5^ |

TKI, tyrosine kinase inhibitor; IS, international scale; UMRD, undetectable minimal residual disease; DMR, deep molecular response
