# Supplementary material for: Clinical Features and T‐Cell Repertoire of Chronic Myeloid Leukemia Patients Who Attempt Discontinuation of Tyrosine Kinase Inhibitors: The ISAC‐TFR Study
Source: Cancer Med. 2025 Aug 11;14(15):e71142. doi: 10.1002/cam4.71142 (PMC12336671; doi:10.1002/cam4.71142)
Supplement: Supplementary file 5 — Data S5: Supporting Information. [file CAM4-14-e71142-s006.docx]

**Table S2. Recent treatment choice for those with relapsed disease, and treatment responses**

| TKIs | CCyR | MMR | MR^4.0^ | MR^4.5^ | UMRD | Total |
| --- | --- | --- | --- | --- | --- | --- |
| Imatinib | 0 | 0 | 0 | 1 | 0 | 1 |
| Dasatinib | 0 | 1 (Relapse after 1 month) | 1 | 4 | 6 | 12 |
| Nilotinib | 0 | 0 | 0 | 0 | 1 | 1 |
| Bosutinib | 0 | 0 | 0 | 1 | 1 | 2 |
| Ponatinib | 1 (T315I) | 0 | 0 | 0 | 3 | 4 |
| Asciminib | 0 | 0 | 1 | 5 | 8 | 14 |
| Total | 1 | 1 | 2 | 11 | 19 | 34 |

TKI, tyrosine kinase inhibitor; CCyR, complete cytogenetic response; MMR, major molecular response; UMRD, undetectable minimal residual disease
