# Supplementary material for: Clinical Features and T‐Cell Repertoire of Chronic Myeloid Leukemia Patients Who Attempt Discontinuation of Tyrosine Kinase Inhibitors: The ISAC‐TFR Study
Source: Cancer Med. 2025 Aug 11;14(15):e71142. doi: 10.1002/cam4.71142 (PMC12336671; doi:10.1002/cam4.71142)
Supplement: Supplementary file 6 — Data S6: Supporting Information. [file CAM4-14-e71142-s003.docx]

**Table S3. Invariant T cell receptor (TCR)s observed in shared TCRα（TRA）and TCRβ（TRB）sequences**

**TRA**

| V gene | J gene | CDR3 | Total reads | CML-13 | CML-28 | CML-38 | CML-43 | CML-53 |
| --- | --- | --- | --- | --- | --- | --- | --- | --- |
| TRAV1-2 | TRAJ33 | CAVIDSNYQLIW | 69 | 0 | 40 | 0 | 29 | 0 |
| TRAV19 | TRAJ49 | CALSETNTGNQFYF | 167 | 57 | 110 | 0 | 0 | 0 |
| TRAV2 | TRAJ33 | CAVDSNYQLIW | 131 | 0 | 0 | 61 | 70 | 0 |
| TRAV21 | TRAJ58 | CAVKETSGSRLTF | 752 | 657 | 0 | 0 | 95 | 0 |
| TRAV21 | TRAJ9 | CAVYTGGFKTIF | 100 | 1 | 11 | 0 | 88 | 0 |
| TRAV23/DV6 | TRAJ57 | CAASIQGGSEKLVF | 76 | 0 | 51 | 25 | 0 | 0 |
| TRAV29/DV5 | TRAJ30 | CAASDGDDKIIF | 85 | 0 | 0 | 32 | 53 | 0 |
| TRAV4 | TRAJ39 | CLVGDNNAGNMLTF | 127 | 0 | 84 | 0 | 43 | 0 |
| TRAV9-2 | TRAJ52 | CALGGTSYGKLTF | 703 | 0 | 684 | 19 | 0 | 0 |
| TRAV9-2 | TRAJ42 | CALSGDGGSQGNLIF | 90 | 0 | 63 | 0 | 27 | 0 |

**TRB**

| V gene | J gene | CDR3 | Total reads | CML-13 | CML-28 | CML-38 | CML-43 | CML-53 |
| --- | --- | --- | --- | --- | --- | --- | --- | --- |
| TRBV11-3 | TRBJ1-1 | CASSLDRGSTEAFF | 131 | 111 | 0 | 0 | 20 | 0 |
